# Insights on the Optical Properties of Poly(3,4-Ethylenedioxythiophene):Poly(styrenesulfonate) Formulations by Optical Metrology

**DOI:** 10.3390/ma10080959

**Published:** 2017-08-17

**Authors:** Argiris Laskarakis, Varvara Karagkiozaki, Despoina Georgiou, Christoforos Gravalidis, Stergios Logothetidis

**Affiliations:** Nanotechnology Lab LTFN, Department of Physics, Aristotle University of Thessaloniki, 54124 Thessaloniki, Greece; vakaragk@physics.auth.gr (V.K.); dgeorgio@physics.auth.gr (D.G.); cgrava@physics.auth.gr (C.G.); logot@auth.gr (S.L.)

**Keywords:** Organic Electronics, Organic Photovoltaics, transparent electrodes, PEDOT:PSS, Spectroscopic Ellipsometry, optical properties

## Abstract

Poly(3,4-ethylenedioxythiophene):poly(styrenesulfonate) (PEDOT:PSS) is among the most widely used polymers that are used as printed transparent electrodes for flexible Organic Electronic (OE) devices, such as Organic Photovoltaics (OPVs). The understanding of their optical properties and the correlation of the optical properties with their electronic properties and metallic-like behavior can lead to the optimization of their functionality as transparent electrodes in multilayer OE device architectures. In this work, we study the optical properties of different PEDOT:PSS formulations by non-destructive Spectroscopic Ellipsometry (SE), from the infrared to the far ultraviolet spectral regions. The optical response of PEDOT:PSS includes an intense optical absorption originated from the conductive part (PEDOT) at lower photon energies, whereas the electronic transition energies of the non-conductive PSS part have been measured at higher photon energies. Based on the different PEDOT:PSS formulations, the optical investigation revealed significant information on the relative contribution of conductive PEDOT and insulating PSS parts of the PEDOT:PSS formulation in the overall optical response, which can strongly impact the final device functionality and its optical transparency.

## 1. Introduction

The polymer-based Organic Electronic (OE) devices, such as organic photovoltaics (OPVs), organic light-emitting diodes (OLEDs), organic thin-film transistors (OTFTs), and sensors, are expected to revolutionize conventional electronics and enable new and smart functionalities to consumer products in applications to energy, buildings, packaging, medicine, etc. [1,2,3,4,5,6,7,8]. Therefore, the fabrication of OE devices on flexible substrates by sheet-to-sheet (s2s) and roll-to-roll (r2r) printing processes is one of the most rapidly expanding sectors of modern science and technology [6,7]. One of the main challenges for printing functional OE devices on flexible plastic substrates is the replacement of the currently used inorganic electrodes, such as indium tin oxide (ΙΤΟ), by solution processable electrodes with tunable optical transparency and electrical properties. Although the inorganic electrodes exhibit good electrical response (low resistivity of ~2 × 10^−4^ Ωcm and relatively high work function of ~4.8 eV), they have several disadvantages, such as high cost, and brittleness against mechanical deformation [3,9,10,11].

One of the most widely used polymers that can be used as transparent electrodes in OE devices is the Poly(3,4-ethylenedioxythiophene):poly(styrenesulfonate) (PEDOT:PSS) [12,13,14,15]. The PEDOT:PSS is a promising π-conjugated polymer that has various technological applications [6,7,13,16,17,18,19,20]. Its major advantages include its tunable electrical conductivity, which facilitates charge transport, and its tunable optical transparency in the visible region, which is essential for light penetration in the device (e.g., in OPVs) or for light emission from the device (e.g., in OLEDs). In addition, it can be solution processed as a continuous film with low surface roughness on rigid and flexible substrates by the use of various lab-scale (as spin coating) and large scale manufacturing methods (as inkjet printing, slot die, and screen printing) [13,21,22,23]. Finally, the electron blocking behavior of PEDOT:PSS can control the direction of current flow in conventional-architecture OPV devices, regardless of the direction of the built-in potential due to the Fermi levels of the electrodes [13,24,25,26,27].

This polymer system consists of a conducting part, PEDOT, which is a low molecular weight polymer that is insoluble and thus difficult to process, and an insulating polymer, PSS, which is a high molecular weight polymer that gives the desirable flexibility and also increases the solubility of the system in water, making the whole system easy to process. The oligomer PEDOT segments are electrostatically attached on the PSS polymer chains [28,29]. The correlation of the optoelectronic properties of PEDOT:PSS formulations, with its structure and the ratio between its conductive and its insulating parts, will contribute significantly to its optimization as an transparent printable electrode for OE devices. Furthermore, the correlation between its optical and electrical properties will enable the optical engineering of the printed OE devices with tunable optical transparency for specific applications. 

In this work, we investigate the optical and electronic properties of different PEDOT:PSS formulations with various compositions and ratios between its conductive (PEDOT) and insulating (PSS) parts, by non-destructive optical metrology in a wide spectral region, from the infrared (IR) to the far ultraviolet (far UV, or fUV) range. The analysis of the measured optical properties of the different PEDOT:PSS formulations can provide significant information on the contribution of the conductive and insulating parts of PEDOT:PSS on its overall optical response. This information can contribute to the tuning of the overall optical transparency of organic electronic devices in which PEDOT:PSS can be implemented as a transparent electrode.

## 2. Results and Discussion

**Figure 1:**
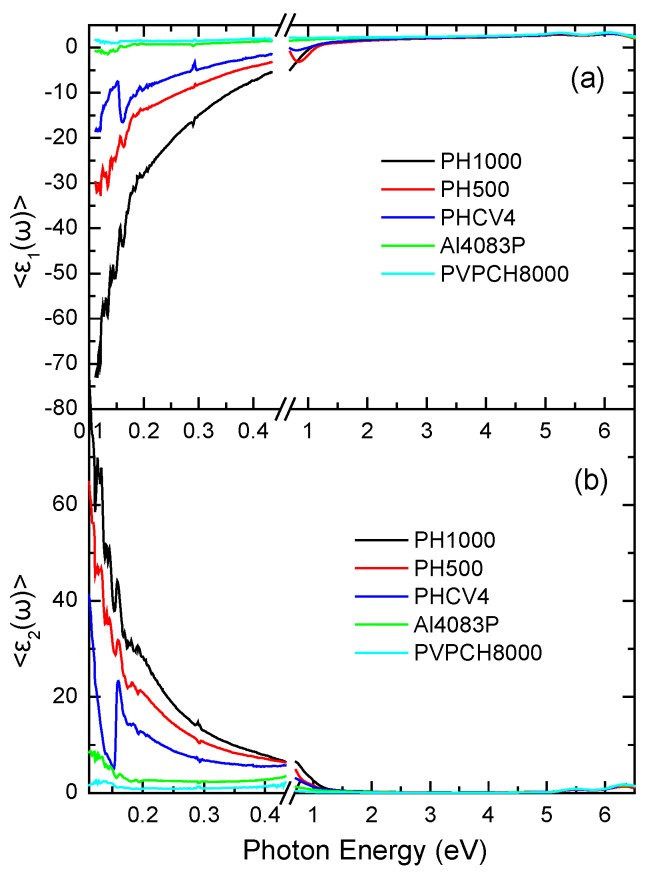
Real (**a**) and imaginary (**b**) parts of the measured pseudodielectric function <$\tilde{\varepsilon }\left(\omega \right)$> of the different poly(3,4-ethylenedioxythiophene):poly(styrenesulfonate) (PEDOT:PSS) formulations in the infrared (IR)-Visible- far ultraviolet (fUV) spectral region.

Figure 1 shows the experimentally measured pseudodielectric function <$\tilde{\varepsilon }\left(\omega \right)$> of different PEDOT:PSS formulations (that were drop-casted on glass substrates), in the IR (900–4000 cm^−1^) and NIR-Vis-fUV spectral regions (0.7–6.5 eV). The measured changes in the <$\tilde{\varepsilon }\left(\omega \right)$> are attributed to the different structure of the different PEDOT:PSS formulations (PH1000, PH500, PHCV4), starting with the more conductive PH1000, and leading to the less conductive formulations, such as PH500, PHCV4, AI4083P, PVPCH8000. The optical response of all of the PEDOT:PSS formulations shows an intense absorption band together with an electronic transition centered at ~1.3 eV. These are attributed to the conductive PEDOT part. From Figure 1, we observe that the optical absorption at the lower energy regions in the IR region is more intense in the case of the more conductive formulations (PH1000, PH500, PHCV4). Moreover, we observe two electronic transitions at the higher photon energies of 5.3 and 6.4 eV, which can be attributed to the π–π* transitions of the benzene rings of the PSS. The optical absorption of all PEDOT:PSS formulations is minimum in the visible spectral region at wavelengths of 400–700 nm (1.7–3.1 eV), indicating the optical transparency of PEDOT:PSS in the visible part of the electromagnetic spectrum, and its functionality as a transparent electrode. The observed differences in the optical properties of the PEDOT:PSS formulations PH1000, PH500 and PHCV4, which have the same PEDOT to PSS ratio (1:2.5), can be attributed to the different additives that have been included in these formulations by the material provider. 

**Figure 2:**
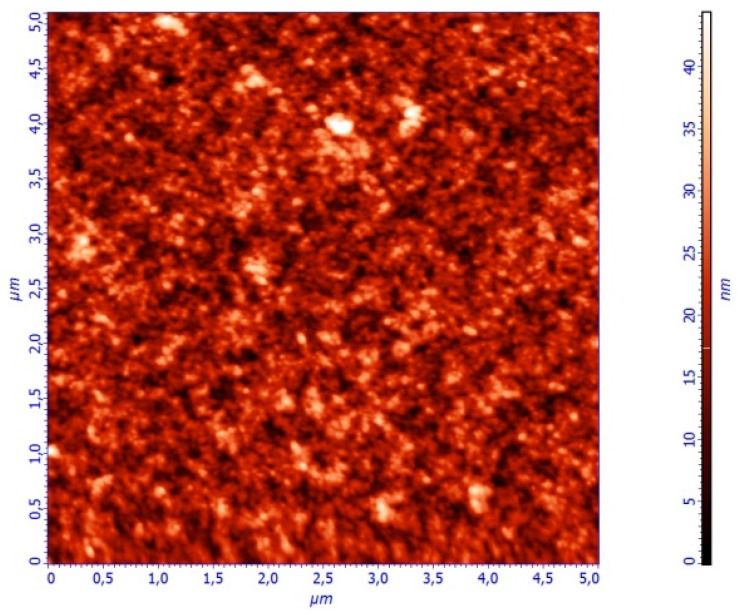
Surface nanotopography of a representative PEDOT:PSS film (formulation PH1000) as measured by AFM. The root mean square (rms) roughness value is measured at 4 nm.

Spectroscopic Ellipsometry is an effective, non-destructive method to determine the pseudodielectric function <$\tilde{\varepsilon }\left(\omega \right)$> and thickness of a large variety of conductive, semiconducting or dielectric films, through the analysis of the measured ellipsometric angles (ψ, Δ) by a suitable theoretical model [30,31]. The analysis of the measured pseudodielectric function <$\tilde{\varepsilon }\left(\omega \right)$> has been realized by the use of a theoretical model that consists of the layer sequence: glass (substrate)/PEDOT:PSS/air (ambient). The optical response of the glass substrates has been determined in advance, and has been used as a reference for the analysis of the <$\tilde{\varepsilon }\left(\omega \right)$> of PEDOT:PSS. Also, the film roughness (root mean square value, or rms) has been measured by Atomic Force Microscopy (AFM) to be in the order of 3 nm in all samples. Figure 2 shows an image of the measured surface nanotopography of a representative PEDOT:PSS film (formulation PH1000), which has a rms roughness of 3 nm. Therefore, the addition of an effective surface layer consisting of PEDOT:PSS and voids in the theoretical model did not provide further accuracy in the calculated optical parameters.

For the determination of the optical response of the PEDOT:PSS films, the measured <ε(ω)> has been modelled by a theoretical model to extract quantitative information on the electronic transitions of the material. This procedure includes the formulation of a theoretical model (which approximates the film architecture and structure of the studied material), and the fitting of the measured <ε(ω)> to this model by using the desired parameters as variables in the numerical analysis. The quality of the numerical analysis fit is determined by the calculation of the root mean square error parameter, which calculates the error value between the measured and theoretical Ψ and Δ values [30,32].

The optical properties of the PEDOT:PSS layer has been described by a combination of the Lorentz–Drude model for the conductive PEDOT and the Tauc Lorentz (TL) model [30,32]. The Lorentz oscillator model has been used in order to describe the interband absorption of PEDOT found at energies ~1 eV. Also, for the analysis of the PSS part, we have used 2 TL oscillators to describe the optical absorptions of the insulating PSS that are found at energies 5.4 and 6.3 eV. Although it has been reported that the optical analysis of PEDOT:PSS films requires the use of an uniaxial anisotropic layer with the optic axis parallel to the surface normal [33], in our work the analysis of the <$\tilde{\varepsilon }\left(\omega \right)$> has been performed by the use of an isotropic model. This has been found to best fit the experimental data from the drop-casted PEDOT:PSS films. 

**Figure 3:**
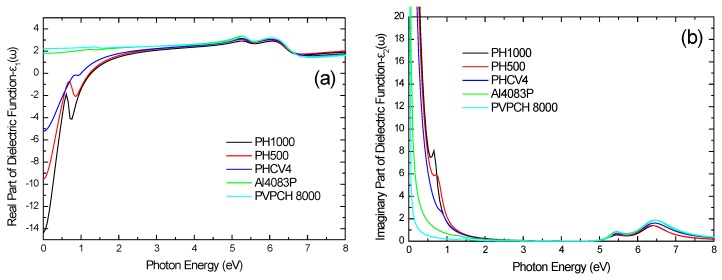
(**a**) Real and (**b**) imaginary parts of the calculated bulk dielectric function of the different PEDOT:PSS formulations in the IR–UV spectral region.

Figure 3 shows the real and imaginary parts of the calculated bulk dielectric function ε(ω) of the different PEDOT:PSS formulations in the extended spectral region from the IR to the fUV. It is clear that the PEDOT:PSS formulations with higher electric conductivity (PH1000, PH500, PHCV4) are characterized by higher contributions at the low energy region, whereas the optical absorptions that correspond to the π–π* transitions of the benzene rings of the PSS are stable for all PEDOT:PSS formulations. 

**Figure 4:**
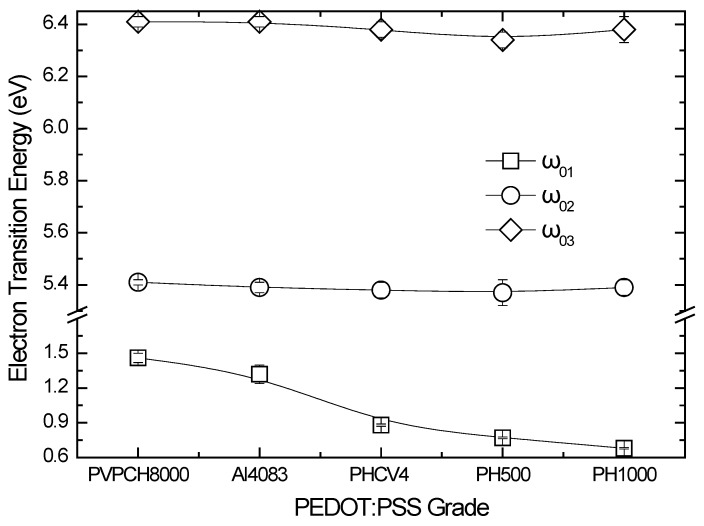
Calculated electronic transition energies of the different PEDOT:PSS formulations.

Figure 4 shows the evolution of the calculated values of the electronic transition energies of the PEDOT and PSS parts for the different PEDOT:PSS formulations. It is clear that in the case of the electronic transition energies that correspond to the insulating PSS part, we observe that these values remain constant for all PEDOT:PSS formulations to 5.4 and 6.4 eV. However, in the case of the electronic transition of the conductive PEDOT part, we observe a reduction from 1.46 eV (for the case of the most insulating PVPCH8000 PEDOT:PSS formulation) to 0.68 eV (for the most conductive PH1000 PEDOT:PSS formulation).

The structure and the morphology of these polymer nanomaterials strongly affect their conductivity. As it has been reported, the morphology of PEDOT:PSS consists of the PEDOT:PSS grain-like particles and of the excess of PSS, which is located at the grain boundaries and on the surface of the film, creating a polymer matrix [16,17,24,28]. The outer layer of the PEDOT:PSS particles is PSS-rich, and the core of the particles is PEDOT-rich. After film formation, PSS acts as a barrier to the conduction of the carriers. The conduction is performed within the grains with hops from one PEDOT segment to another according to the hopping model [28,34].

**Figure 5:**
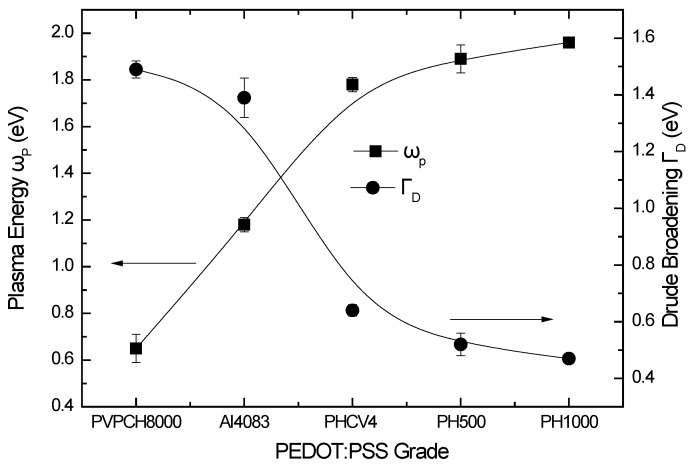
Calculated plasma energy (ω_p_), and Drude broadening (Γ_D_) values of the different PEDOT:PSS formulations.

Figure 5 shows the evolution of the calculated plasma energy ω_p_ and the Drude broadening. The plasma energy is relative to the free carrier density N, and it is described by the relation [30,31]:

(1) $$\omega_{p}=\sqrt{\frac{{\mathrm{Ne}}^{2}}{\varepsilon_{0}m^{*}}}$$

where ε_0_ is the absolute permittivity, m* is the effective carrier mass, and e is the electric charge. The calculated plasma energy values are found to increase in the case of the conductive PEDOT:PSS formulations (PVPCH8000, PH500, PH1000), starting from 0.65 eV for the PVPCH8000 formulation, to 1.96 eV for the PH1000 formulation. On the contrary, the Drude broadening term that is related to the long-range order of the material reduces from 1.49 for the PVPCH8000 formulation to 0.47 for the PH1000 formulation. Therefore, the conductive PEDOT:PSS formulations are characterized by a higher number of charge carriers.

The above are justified by the study of the broadening parameter (or damping factor) Γ_D_ (=ħ/τ_D_), which is the inverse of the relaxation time τ_D_, and depends on the phonon contribution and on microstructural properties of the material, that include static impurities, defect density, grain boundary, grain size etc. As it can be seen from Figure 5, the Γ_D_ values are reduced for the case of the more conductive PEDOT:PSS formulations, leading to higher relaxation times of the charge carriers, which are influenced by the existence of grain boundaries, and structural defects within the PEDOT:PSS structure [16,35].

The enhancement of the carrier mobility is mainly attributed to the delocalization of charge carriers, which is correlated to the increase in packing density of the PEDOT:PSS nanoparticles, due to the reduction of the excess PSS [36]. This results to a formation of a three-dimensional conducting network that improves the macroscopic carrier transport. The reduction of the PSS on the less conductive PEDOT:PSS formulations can be supported by the measurement of the surface energy by contact angle measurements. The less conductive PEDOT:PSS formulations (such as PVPC8000) are characterized by a higher surface energy (70.7 mN/m) than the more conductive PH1000 PEDOT:PSS formulation (53.9 mN/m). Therefore, the hydrophilic PSS part has a lower volume fraction at the PEDOT:PSS formulation of higher conductivity (PH1000).

**Figure 6:**
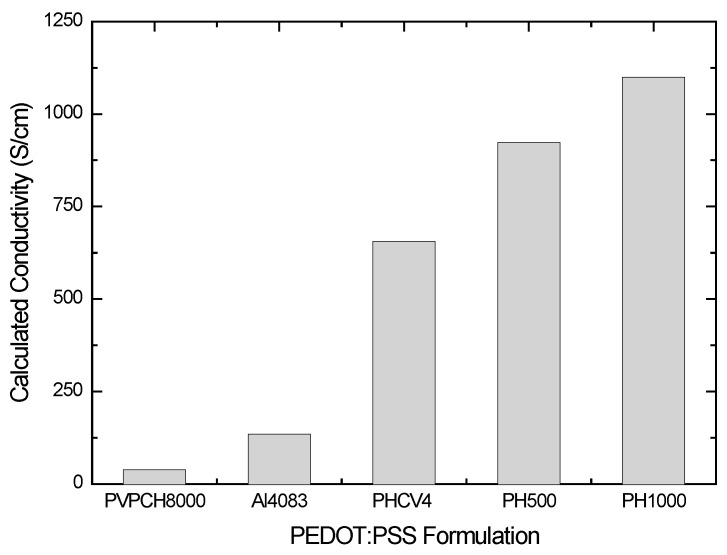
Calculated conductivity values by the modelling of the measured <$\tilde{\varepsilon }\left(\omega \right)$> by Spectroscopic Ellipsometry (SE) of the different PEDOT:PSS formulations.

Figure 6 shows the calculated values of conductivity that have been calculated by the Drude model parameters (Equation (1)) for the different PEDOT:PSS formulations. The determined conductivity values are higher in the case of the PEDOT:PSS formulations, with a higher amount of PEDOT parts per PSS parts (higher PEDOT:PSS ratio), as in the case of PH1000, PH500, and PHCV4. The increase in the calculated conductivity can be attributed to the increase of the average domain size of PEDOT particles in combination with the reduction of the insulating PSS barriers between the PEDOT grains (less energy barriers) [37]. Furthermore, the calculated conductivity values from the analysis of the measured pseudodielectric function (optical conductivity) appear to be higher than the reference conductivity values of the PEDOT:PSS formulations (reference conductivity value of PH100 is 1000 S/cm, according to the manufacturer). This discrepancy can be attributed to the fact that the calculated conductivity values are more representative of the local conductivity in PEDOT-rich regions. On the contrary, the measurement of the electrical conductivity (e.g., by four point probe technique) is representative of the global conductivity of the sample in which the more resistive PSS contributes [26]. 

## 3. Materials and Methods 

The PEDOT:PSS films are commercially available as an aqueous colloidal solution, whereas during the casting of PEDOT:PSS films from solution, their structure is maintained in the film. In this work, thin films of PEDOT:PSS were drop-casted on quartz glass substrates, and the thickness of the drop-casted PEDOT:PSS layers was ~3 μm. After the coating, the samples were heated for 90 s at 100 °C in order to remove the solvent.

Five different aqueous dispersions of commercially supplied PEDOT:PSS materials obtained from Heraeus were used. These formulations are the Clevios PH1000 (1:2.5), PH500 (1:2.5), PHCV4 (1:2.5), Al4083 (1:6), and PVPCH8000 (1:20). These PEDOT:PSS formulations have been used as transparent electrodes in several OE devices, such as OPVs and OLEDs [6,13,25,26,27,28,35,38,39,40,41]. The intrinsic properties of each PEDOT:PSS formulation defines its functionality within the OE device architecture [19,38]. The PH1000, PH500, and PHCV4 are among the most conductive commercially suppled formulations, and they can be used as either cathode or anode electrodes in solution processed semi-transparent OPV devices of normal (substrate/ITO/PEDOT:PSS/photoactive/cathode) or inverted architecture (substrate/ITO/electrode/photoactive/PEDOT:PSS/anode electrode). The conductivity values of these formulations are reported in the range of: ~1000 S/cm for the PH1000, in the range of ~400–600 S/cm, for the PH500, and in the range of 400 S/cm for the PHCV4 (usually by the addition of organic solvents such as dimethyl sulfoxide or diethylene glycol [13,25,26,35,36,38,42]. The Al4083 and PVPCH8000 formulations are characterized by conductivities in the range of 10^−3^–10^−4^ S/cm and 10^−5^ S/cm, respectively, and they are used as hole injection layers in OE devices [13,18,25,38,43]. PEDOT:PSS can significantly enhance the hole extraction towards the cathode electrode in inverted OPV device architectures, whereas it can improve the OPV performance through the planarization of the underlying ITO layer, and it can increase the open-circuit voltage V_oc_ of OPVs through the reduction of the dark current of the OPV device [25,27,38]. 

The optical and electronic properties were measured by the use of non-destructive Spectroscopic Ellipsometry (SE) in an extended spectral region by a Fourier Transform IR Spectroscopic Ellipsometer (FTIRSE) working on the IR spectral range (900–4000 cm^−1^) and by a Phase Modulated Spectroscopic Ellipsometer (Horiba Jobin-Yvon) working at the near IR-visible-far ultraviolet spectral region (0.7–6.5 eV). The setup of the FTIRSE system has been described in detail elsewhere [44,45,46]. The FTIRSE and SE units use a light beam generated by SiC rods and an Xe lamp, respectively, that passes through the polarizer and the modulator of the system in order to focus on the sample under study, with an angle of incidence of 70° [44,45,46]. After this reflection on the sample surface, mirrors and lenses focus the reflected light beam on the detection head, which consists of the analyzer and the detector. 

For the calculation of the conductivity σ = 1/ρ where ρ is the resistivity, we have used the determined parameters from the Drude model analysis of the measured <$\tilde{\varepsilon }\left(\omega \right)$> (plasma energy ω_p_ and the Drude broadening Γ_D_) by using the following relation [32,47,48]:

(2) $$\rho =0.007435\cdot \frac{\Gamma_{D}}{\omega_{P}^{2}}$$

The surface nanomorphology has been investigated by AFM with a NTEGRA Scanning Probe Microscope (NT-MDT, Moscow, Russia). The tapping mode was utilized for better image acquisition, using rectangular Si cantilevers with a nominal tip curvature of 10 nm. Finally, the surface energy of the various PEDOT:PSS formulations were measured by contact angle (CA) technique, which were performed with an optical contact angle and surface tension meter CAM200 (KSV Instruments Ltd., Helsinki, Finland). The liquid was water, and the drop volume was 5 μL, with a surface tension of 72.8 mN m^−1^. The contact angles were converted into surface energy values using the relation outlined by Chibowski et al. [49].

## 4. Conclusions

In summary, we have investigated the metallic-like behavior of different PEDOT:PSS formulations by modelling their optical properties in the IR–fUV spectral region by the use of non-destructive SE. The PH1000, PH500, and PHCV4 formulations are characterized by higher metallic contribution at the IR photon energies as a result of the increased charge carrier density, and the higher ratio of the conductive PEDOT part in the material structure. In addition, we have determined the electric conductivity of the PEDOT:PSS formulations through the analysis of the optical properties by non-destructive SE measurements. This information can contribute to the understanding of the optical investigation of multilayered organic electronic devices in which PEDOT:PSS is used as an transparent electrode and buffer layer. This research also denotes the importance of optical metrology methods in the optimization of the printing processes for the fabrication of OE devices on flexible substrates.
